# Prostanoid receptor EP1 and Cox-2 in injured human nerves and a rat model of nerve injury: a time-course study

**DOI:** 10.1186/1471-2377-6-1

**Published:** 2006-01-04

**Authors:** Pascal F Durrenberger, Paul Facer, Maria A Casula, Yiangos Yiangou, Roy A Gray, Iain P Chessell, Nicola C Day, Sue D Collins, Sharon Bingham, Alex W Wilson, David Elliot, Rolfe Birch, Praveen Anand

**Affiliations:** 1 Peripheral Neuropathy Unit, Imperial College London, Area A, Ground Floor, Hammersmith Hospital, London W12 0NN, UK; 2 Neurology & GI CEDD, GlaxoSmithKline, Harlow, UK; 3 St Andrew's Centre, Broomfield Hospital, Chelmsford, UK; 4 Peripheral Nerve Injury Unit, Royal National Orthopaedic Hospital, Stanmore, UK

## Abstract

**Background:**

Recent studies show that inflammatory processes may contribute to neuropathic pain. Cyclooxygenase-2 (Cox-2) is an inducible enzyme responsible for production of prostanoids, which may sensitise sensory neurones via the EP1 receptor. We have recently reported that while macrophages infiltrate injured nerves within days of injury, they express increased Cox-2-immunoreactivity (Cox-2-IR) from 2 to 3 weeks after injury. We have now investigated the time course of EP1 and Cox-2 changes in injured human nerves and dorsal root ganglia (DRG), and the chronic constriction nerve injury (CCI) model in the rat.

**Methods:**

Tissue sections were immunostained with specific antibodies to EP1, Cox-2, CD68 (human macrophage marker) or OX42 (rat microglial marker), and neurofilaments (NF), prior to image analysis, from the following: human brachial plexus nerves (21 to 196 days post-injury), painful neuromas (9 days to 12 years post-injury), avulsion injured DRG, control nerves and DRG, and rat CCI model tissues. EP1 and NF-immunoreactive nerve fibres were quantified by image analysis.

**Results:**

EP1:NF ratio was significantly increased in human brachial plexus nerve fibres, both proximal and distal to injury, in comparison with uninjured nerves. Sensory neurones in injured human DRG showed a significant acute increase of EP1-IR intensity. While there was a rapid increase in EP1-fibres and CD-68 positive macrophages, Cox-2 increase was apparent later, but was persistent in human painful neuromas for years. A similar time-course of changes was found in the rat CCI model with the above markers, both in the injured nerves and ipsilateral dorsal spinal cord.

**Conclusion:**

Different stages of infiltration and activation of macrophages may be observed in the peripheral and central nervous system following peripheral nerve injury. EP1 receptor level increase in sensory neurones, and macrophage infiltration, appears to precede increased Cox-2 expression by macrophages. However, other methods for detecting Cox-2 levels and activity are required. EP1 antagonists may show therapeutic effects in acute and chronic neuropathic pain, in addition to inflammatory pain.

## Background

Tissue damage induces an inflammatory response including the production of prostaglandins (PGs) such as PGE_2_, which activate the EP1 receptor expressed by sensory fibres. PGs produced in the spinal cord may also play an important role in the development of hypersensitivity following peripheral nerve injury [1]; PGs generated by Cox-2 in the spinal cord have been shown to contribute to the maintenance of hyperalgesia [2].

The enzymes involved in the production of PGs are cyclooxygenases (Cox) of which Cox-1 was at first thought to be the only enzyme present. Subsequently, it was found that Cox activity could be induced by inflammatory cytokines, suggesting the existence of a second isoform. This was confirmed by the isolation of a second cyclooxygenase gene encoding Cox-2 [3]. The classical view that Cox-1 was constitutive and that Cox-2 was exclusively a pro-inflammatory inducible enzyme [4] was challenged since both isoforms are present in different tissues and sites of inflammation, and induced differentially [5,6]. Cox-2 protein is upregulated in a number of non-neuronal cell types such as macrophages, human monocytes, synoviocytes, and microglia in CNS inflammation [7,8]. Data indicate that Cox-2 is strongly involved in different processes of central nervous modelling and regulated by different signalling pathways. The explicit roles of the constitutive enzyme in the pain and inflammatory processes remains to be fully determined [9]. Evidence that prostanoids could sensitise the peripheral nerve terminals [10] has triggered new research in the Cox enzymes involved in the biosynthesis of PGs to develop inhibitors (Coxibs) of potential therapeutic value.

The contribution of prostanoids such as PGE_2 _or PGE_2α _in inflammatory processes [11] and in pain modulation has well been defined [12,13] and reviewed [14]. PGE_2 _signals via a transmembrane G-protein coupled receptor (EP), of which four types have been identified (EP1-4) [13,15]. EP1 receptor stimulation mediates increases in intracellular calcium ions (Ca^2+^), facilitating neurotransmitter release [16,17]. EP1 receptor involvement in pain mechanisms has been described in animal studies [18,19]. EP receptor antagonists have provided evidence of a role for EP receptors in reducing hyperalgesia and allodynia in rodents [20]. Localisation studies have revealed that EP1 mRNA is expressed in rat DRG neurones [21-23]. A recent study demonstrated that PGE_2_, via the EP1 receptor, contributed to human visceral pain hypersensitivity [24]. The emerging general consensus of animal and human studies identifies the EP1 receptor as a selective target of therapeutic value, of similar analgesic effect as non-steroidal anti-inflammatory drugs (NSAIDs), but with fewer potential side effects [18].

Activation of immune-like glial cells such as astrocytes or microglia has been reported in numerous conditions, and may contribute to hyperalgesia, mechanical allodynia or chronic inflammatory pain in animal models. Microglia are phagocytic, cytotoxic and antigen-presenting cells that upon activation are involved in a pattern of cellular responses, including proliferation, recruitment to the site of injury and increased expression of immunomolecules [25]. Glial activation can be induced by substances released from neurones such as PGs, nitric oxide, fractalkine, substance P, excitatory amino acids and adenosine 5'-triphosphate (ATP) [26], and in turn, result in the release of numerous inflammatory agents such as cytokines, growth factors, kinins, purines, amines, prostanoids and ions [27]. These inflammatory agents have been shown to activate and/or enhance the sensitivity of primary afferents and spinal cord neurones, and thus glial activation may play a role in nociceptive processing [28-31]. However, some studies report lack of correlation of neuropathic pain behaviour with levels of microglial activation in animal models [32].

The aim of this study was to investigate the time-course of key neuronal-inflammatory interactions in injured human nerves and DRG, and in the CCI rat model. Macrophage/microglia-like cells, Cox-2 and EP1 receptor levels were studied, using immunocytochemistry and Western blotting.

## Methods

### Human tissue

Fully informed consent was obtained for all tissues, which were collected with approval of the Local Ethics Committee. Injured human nerve specimens (proximal and distal to site of injury) were obtained during surgery for brachial plexus repair [n = 11; acute (< 21 days), n = 5, 5 males, age range 20–66 years; chronic (> 21 days) n = 6, 4 males and 2 females, age range 24–35 years] and avulsed DRG [n = 11; acute (< 21 days) n = 5, 5 males, age range 18–39 years; chronic (> 21 days), n = 6, 5 males and 1 female, age range 21–39 years]. Painful human distal limb neuromas were obtained during surgery (n = 12; 9 males and 3 females; age range 26–63 years; injury duration from 9 days to 12 years) – the mean pain scores on a visual analogue scale (VAS) were all > 4 out of 10 at the time of surgery, and patients reported this or a higher level of pain usually continuously since the time of injury. Uninjured control human nerve tissue (n = 9, 5 males and 4 females, age range 39–77 years) was obtained during surgery for limb amputation for non-neurological tumours from patients with no neurological symptoms or signs. Control, post-mortem DRG (n = 7, 2 males and 5 females, age range 34–88) were obtained from Netherlands Brain Bank with a post-mortem delay of less than 12 h. Tissues were snap frozen in liquid nitrogen and stored at -70°C until use.

### Rat tissue

Adult male Sprague-Dawley rats (n = 32; 200–250 g) were used in this study. CCI animals (n = 16) had the left sciatic nerve loosely ligated with chromic gut sutures to cause a constriction injury. Control, sham-operated animals (n = 16) underwent identical surgical procedures but without nerve constriction. In brief, under isoflurane anaesthesia, the common left sciatic nerve was exposed at mid thigh level. Four loose ligatures of chromic gut (4.0) were tied loosely around the nerve with a spacing of 1 mm between each. The wound was then closed and secured with suture clips. The surgical procedure was identical for the sham-operated animals except the sciatic nerve was not ligated. CCI-induced decrease in mechanical paw withdrawal threshold was measured using an algesymeter [33]. To determine threshold, an increasing weight was applied to the dorsal surface of the left and right hindpaw until the rat attempted to withdraw the paw. To study glial activation and Cox-2 expression, animals were sacrificed on days 4, 21, and 30 (n = 4 in each experimental group and for each time point) and tissues harvested. Left and right sciatic nerve (nerve tissue from 4 days post-operation only was available for this study), and lumbar spinal cord were collected. Tissues were snap frozen in 2-methyl butane cooled in liquid nitrogen and stored at -70°C until use. All procedures involving the use of animals were approved by the UK Home Office and were carried out in accordance with the requirements of the project licence.

### Immunocytochemistry

Tissues were supported in optimum cutting tissue (OCT) medium (Raymond A Lamb Ltd, Eastbourne, UK) to allow best orientation (transverse for spinal cord, longitudinal for nerve). Frozen sections (10 μm thick) were collected onto poly-L-lysine (Sigma, Poole, UK) coated glass slides and post-fixed in freshly prepared 4% w/v paraformaldehyde in 0.15 M phosphate buffered saline (PBS) for 30 min. Endogenous peroxidase was blocked by incubation in industrial methylated spirit (IMS) containing 0.3% w/v hydrogen peroxide. After rehydration, sections were incubated overnight with primary antibody. The primary antibodies for EP1 were polyclonal rabbit anti-human EP1 receptor antibody (1:500, Cayman Chemical, Bingham, UK) and polyclonal rabbit anti mouse EP1 receptor antibody (1:500, Alpha Diagnostic International, San Antonio, Texas, USA), which showed similar results (results shown for Cayman antibody only). Other primary antibodies used included monoclonal anti-Cox-2 antibody (1:250, Transduction Laboratories, Cowley, UK, Clone33), monoclonal mouse anti-CD68 as a well-established marker of human macrophages [34], described in numerous previous publications (1:500, Dako, Ely, UK, Clone EBM11), mouse anti-rat CD11b antibody as a macrophage/microglial marker in rats (1:1000, Serotec, Kidlington, UK, CloneMRC OX42), a monoclonal mouse antibody to the neurofilament phosphorylated and non-phosphorylated 200 kDa molecular forms (1:50000, Sigma Laboratories, Saint-Louis, MI, USA, Clone N52) and a rabbit polyclonal antiserum antibody cocktail to neurofilaments (1:5000, Affinity Research Products Limited, Exeter, UK). The rat microglial marker (OX42/CD11b) previously showed similar levels of expression in CCI rat tissue as with another microglial marker ([^3^H] (R) PK11195) [35]. Sites of primary antibody attachment were revealed using nickel-enhanced, avidin-biotin peroxidase (ABC – Vector Laboratories, Peterborough, UK) as formerly described [36]. Sections were counter-stained for nuclei in 0.1% w/v aqueous neutral red, dehydrated and mounted in xylene-based mountant (DPX; BDH/Merck, Poole, UK), prior to photomicrography. Negative controls without primary antibody were incubated with normal rabbit or mouse serum. EP1 peptide antigen from GlaxoSmithKline/Cayman Chemicals was used at 10^-1 ^to 10^-6 ^mg/ml and pre-incubated with anti-EP1 (Cayman) at 1/5000 on a control DRG and at 1/2000 on an acute injured DRG. Both control and injured DRG showed reduced staining at high (10^-1 ^– 10^-2 ^mg/ml) concentrations of peptide antigen, compared to sections stained in the presence of antibody alone.

Immunoreactive cells were quantified by computerized image analysis (Seescan Cambridge, UK). Analogue images were captured via video link to an Olympus BX50 microscope and converted into a digital monochrome image by the computer. The grey-shade detection threshold was set at a constant level to allow detection of positive immunostaining and the area of highlighted immunoreactivity was expressed as a % area of the field scanned. One section per nerve specimen was image-analysed at the stated optimum dilution (this followed a series of 5 sections immunostained with 5 different antibody dilutions). The visual fields were selected at random, avoiding edges of the section. The area was 442 μm × 332.8 μm. Three to five fields per tissue section were scanned and the mean value was used in subsequent statistical analysis. For DRG, the intensity of immunoreaction of sensory neurones was assessed in blinded fashion by two observers at two dilutions of antibodies and given a mean score (vision inspection scale: 0 = no immunoreaction; 1 = weak; 2 = medium; 3 = strong). DRG neurones were counted in the entire section (mean 54.58; range 34–87) per DRG.

### Western blotting

Brachial plexus nerves from 4 controls and 8 injured patients (3 acute and 5 chronic injured) were available for Western blotting. Note that only 3 samples for the acute group were available (10, 15 and 17 days of injury duration) and represent the latter stage of the "acute" phase. Nerve extracts and mouse macrophage control extracts (Transduction Labs, Cowley, UK) were processed for Western blotting as described [37]. Briefly, non-specific antibody binding sites were blocked by incubating the strips in 5% (w/v) non-fat dried milk in a solution of PBS containing 0.1% (v/v) Tween 20 for 1 h. Primary antibody incubation was 2 h or overnight in block buffer (Cox-2 at a titre of 1:2000). After washing, sites of attachment of primary antibodies were detected using immunoperoxidase reagents (Vector Laboratories, Peterborough, UK). Immunoreactivity using chemiluminescence was visualised on Hyperfilm film after treatment with Electrophoresis Chemiluminescence (ECL)-plus Western blotting detection system (Amersham Biosciences Ltd, Little Chalfont, UK). Optical density readings of each Hyperfilm were taken using a Digit-X densitometer (X-Ograph Ltd, Tetbury, UK) evenly illuminated on a photography viewer. Background readings were determined by measuring optical density outside the sample lanes. After subtraction of background, the mean of three consecutive readings of protein immunoreactivity at the 70-kDa positions for each sample lane was obtained.

### Statistical analysis

Descriptive statistics were generated using Microsoft Excel 2000 for Windows (Microsoft, Redmond, WA, USA) and GraphPad Prism version 3.00 for Windows (GraphPad Software, San Diego California, USA). Group differences were assessed using a nonparametric test, the Mann-Whitney U test (one-tailed), in GraphPad Prism. Statistical significance was considered at P < 0.05.

## Results

### Human brachial plexus nerves and DRG

**Figure 1:**
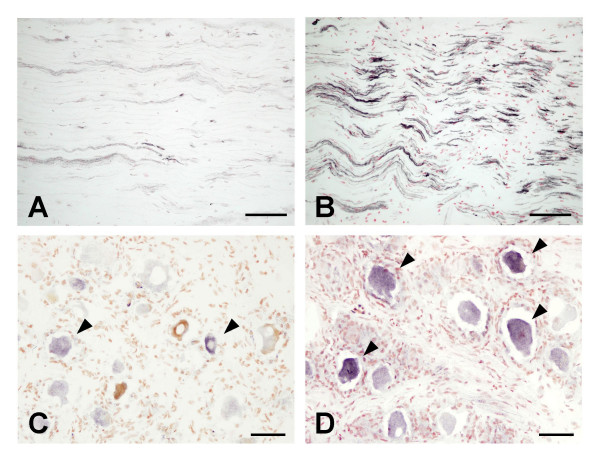
**EP1 immunoreactivity in human injured brachial plexus nerves and avulsed DRG. **EP1-IR was observed in nerve fibres in control nerves (A) and appeared more intense in injured nerves (B). EP1-IR was detected mainly in some small/medium diameter neurones (arrowheads) of control (C) and more so in injured human DRG (D). Scale bars = 50 μm.

Both EP1 antibodies showed immunoreactivity in nerve fibres (Figure 1A), which appeared more intense in acute injured nerves (Figure 1B). The results of image analysis are given below. In human DRG, EP1 immunoreactivity (EP1-IR) was detected in small/medium diameter neurones of control (Figure 1C) and injured human DRG (Figure 1D). A significant increase of intensity was observed using the visual inspection scale in DRG sensory neurones after injury in the acute group (surgery delay < 21 days; 3.5 ± 0.34) compared to control DRG (2.57 ± 0.20, p = 0.02). Chronic injured DRG were not significantly different (2.83 ± 0.17).

**Figure 2:**
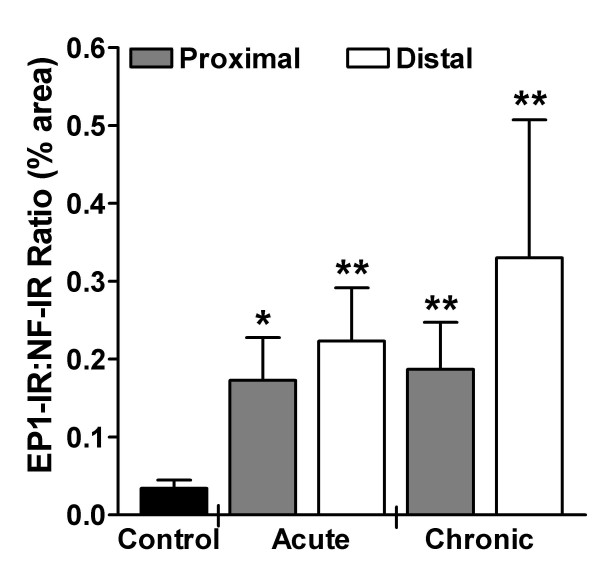
**EP1-IR:NF-IR ratio in human proximal and distal injured nerves**. Significant increases of EP1-IR:NF-IR ratios were observed in human nerves proximal and distal to injury when compared to uninjured nerves. * p < 0.03; ** p < 0.01.

EP1 and NF-immunoreactive nerve fibres were quantified by image analysis (% immunopositive area) in injured and control nerves and expressed as the ratio EP1:NF (Figure 2). EP1-IR:NF-IR ratio was significantly higher in injured acute proximal (0.17 ± 0.05; n = 5; p < 0.03) and distal (0.22 ± 0.69; n = 5; p < 0.01) and injured chronic proximal (0.19 ± 0.06; n = 6; p < 0.01) and distal (0.33 ± 0.18; n = 6; p < 0.01) nerves compared to controls (0.03 ± 0.01; n = 5).

**Figure 3:**
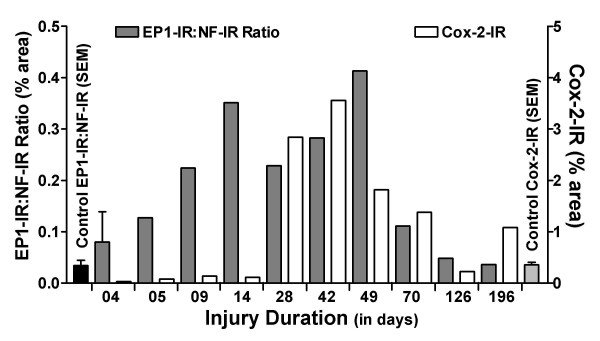
**EP1-IR:NF-IR ratio and Cox-2-IR in proximal brachial plexus injured nerves – time course. **The time course of EP1-IR:NF-IR ratio and Cox-2-IR showed that EP1 preceded Cox-2-IR increased levels.

A time course analysis of EP1-IR:NF-IR ratio and Cox-2-IR in human brachial plexus nerves showed that EP1 expression preceded Cox-2-IR increased levels (Figure 3). Similar results were found with both EP1 markers.

**Figure 4:**
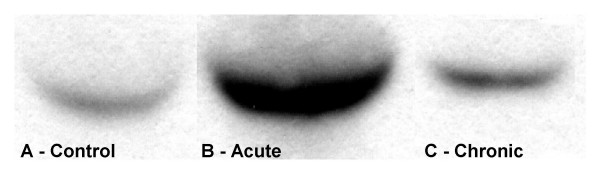
**Cox-2 70-kDa band by Western blots. **Cox-2 70-kDa band in control nerve (A), acute injured nerve (B) and chronic injured nerve (C). The Cox-2 70 kDa band was clearly more prominent in the acute nerves.

A 70 kDa Cox-2 band was observed in mouse macrophage control and human nerve extracts, which was clearly more prominent in the acute nerves (Figure 4). The optical density of the Cox-2 70kDa band was significantly increased (p = 0.02) in the acute group (1.41 ± 0.04, n = 3) compared to control nerves (0.55 ± 0.09, n = 4). No statistical significant difference was detected for the chronic group (0.61 ± 0.09, n = 5).

### Human painful neuromas

**Figure 5:**
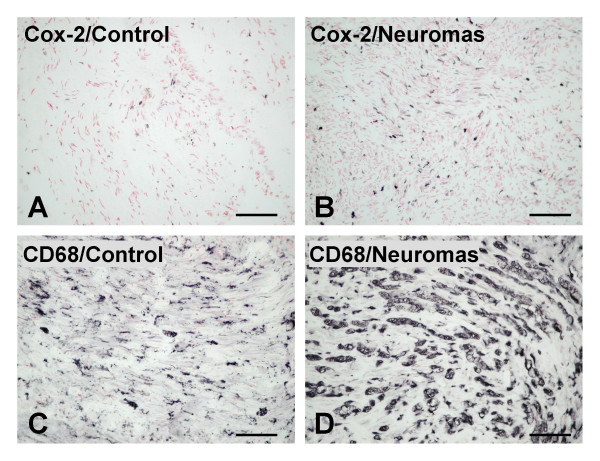
**Cox-2 and CD68 immunoreactive cells in uninjured nerves and human neuromas. **Very few scattered Cox-2 immunoreactive cells were found in uninjured human nerve tissue (A) compared to human neuromas (B). CD68 immunoreative cells were of similar morphology and distribution as Cox-2 and were observed with greater abundance in injured nerve (D) than in control (C). Magnification bars = 50 μm.

Few, scattered microglial/macrophage-like, Cox-2 immunoreactive cells were found throughout the uninjured human nerve tissue (Figure 5A). Similar cells, but with more abundance were observed in the painful neuromas (Figure 5B). Immunostaining for CD68 showed cells with similar morphology and distribution to Cox-2 in controls (Figure 5C), with an increase in neuromas (Figure 5D.).

**Figure 6:**
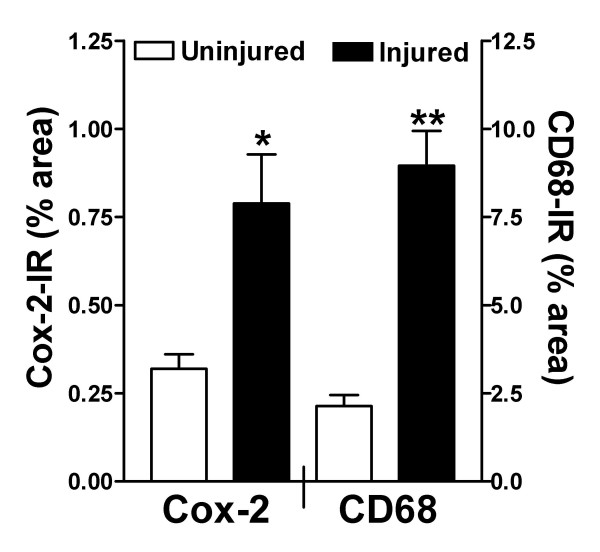
**Cox-2-IR and CD68-IR in human painful neuromas. **Cox-2-IR and CD68-IR (in % area) are significantly increased in injured nerves compared to control nerves. * p < 0.002; ** p < 0.0001.

Image analysis showed Cox-2-IR (in % area) to be significantly greater in human neuromas (0.79 ± 0.14; n = 12; p = 0.0022) than in controls (0.32 ± 0.04; n = 13; Figure 6). A similar increase was found with the macrophage marker CD68. CD68 immunoreactivity (CD68-IR) was significantly increased in human limb neuromas (8.96 ± 0.99; n = 12; p < 0.0001) compared to the control group (0.32 ± 0.04; n = 13).

**Figure 7:**
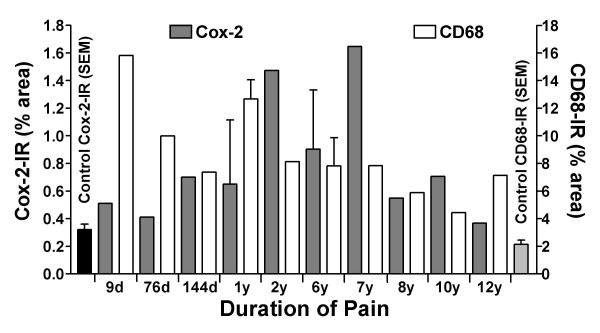
**Cox-2-IR and CD68-IR in human neuromas – time course. **Rapid increase of CD68 immunoreactive cells was observed, whilst Cox-2-IR was only apparent from 2 to 3 weeks as previously described and remained persistent over many years.

A time course analysis of injury duration (time elapsed between the injury and the removal of the neuroma) demonstrated an immediate increase in CD68-IR, which remained above control levels during the entirety of the time course (Figure 7). Cox-2-IR increases, as previously described, were only apparent from 2 to 3 weeks after injury [35], and persistent for years.

Few nerve fibres showed some EP1 immunoreactivity but fibres were too sparse to show significant statistical difference.

### CCI rat model studies

Unilateral constriction injury to the sciatic nerve resulted in a reduction in paw withdrawal threshold ipsilateral to the nerve injury, usually evident at 9 days and maintained until after 30 days post-operation (in these rats, at 30 days post-operation: CCI, 88.12 ± 7.04 g; n = 8, sham 127.5 ± 75.13 g; n = 8). Following behavioural testing on day 04, 21 and 30, the animals were humanely sacrificed and tissues prepared for immunocytochemistry. Sham operation had no significant effect on paw withdrawal threshold compared to basal levels.

**Figure 8:**
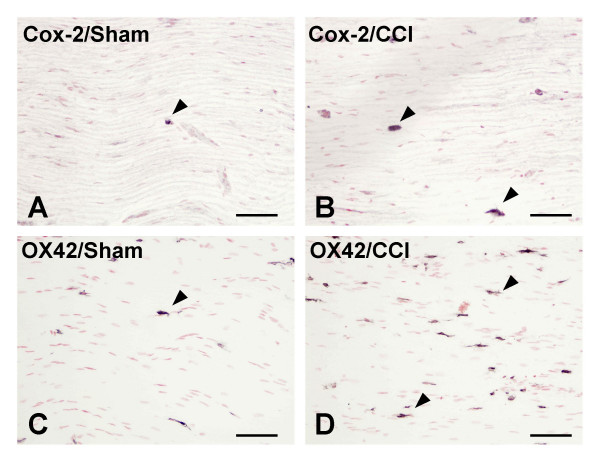
**Cox-2 and OX42 immunoreactive cells in sham and CCI rat nerve at 4 days post operation. **Very few Cox-2 immunoreactive cells (arrowheads) were observed in the left sciatic nerve of sham-operated (A) compared to CCI (B). Similarly, sham-operated nerves (C) showed much less macrophages in the sciatic nerve than CCI nerves (D). Scale bars = 50 μm.

**Figure 9:**
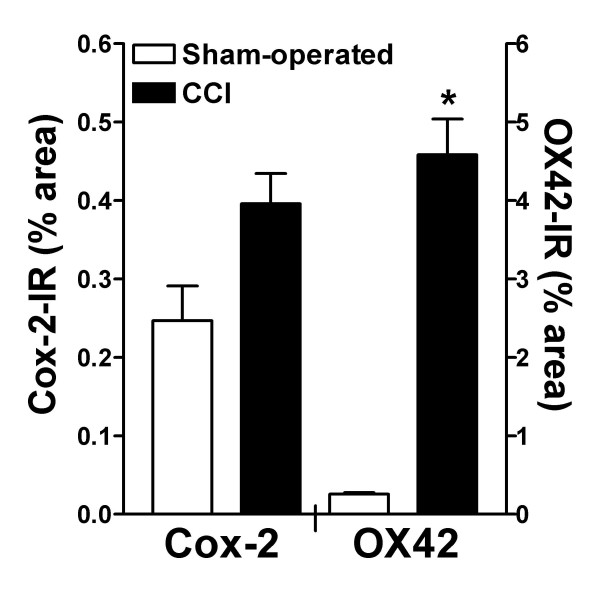
**Cox-2-IR and OX42-IR in sham-operated and CCI lesioned rat nerve at 4 days post-operation. **At 4 days post-operation, OX42-IR was significantly increased in lesioned nerves compared to sham-operated nerves whilst Cox-2-IR was not significantly increased. * p < 0.015.

In rat nerve, Cox-2-IR and CD68-IR were found in cells similar to those seen in human nerve tissue as previously described [35]. Few scattered Cox-2 positive cells were seen within the nerve fascicles in sham-operated or control nerves. In CCI nerve, these cells appeared more numerous proximal to the injury site, but this was not statistically significant at 4 days and showed only a trend (p = 0.057); in our previous study, we have shown a significant increase at 40 days post-surgery [35]. Antibodies to the rat macrophage/activated microglia marker (CD11b-OX42) showed few positively stained cells in sham-operated or control nerves (Figure 8), and an abundance of positively stained cells in day 4 post CCI-lesioned nerves (n = 4, 4.58 ± 0.46) compared to sham-operated nerves (n = 4, 0.26 ± 0.02; p < 0.001, Figure 9).

**Figure 10:**
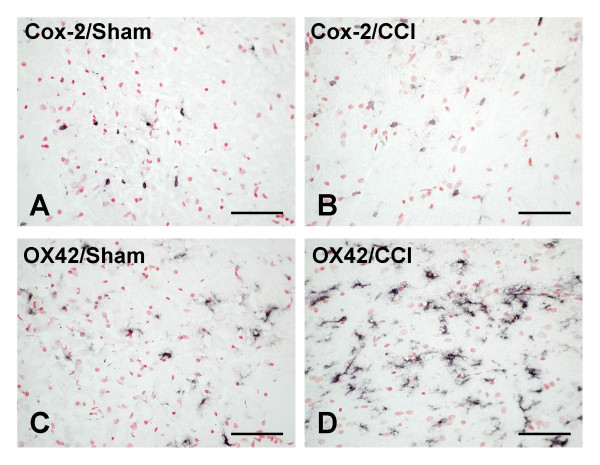
**Cox-2 and OX42 immunoreactive cells in the superficial dorsal horn of rat spinal cord (Laminae I-II). **At 4 days post-operation, Cox-2 immunoreactive cells were more abundant in controls (A) compared to CCI (B), whilst few OX42 immunoreactive cells were seen in the superficial dorsal horn of sham-operated spinal cord (C) compared to CCI (D). Magnification bars = 50 μm.

In the spinal cord of sham-operated rats at all time points, small, microglial-like Cox-2-immunoreactive cells, with some fine processes, were scattered throughout the grey matter and the white matter (Figure 10A). These cells tended to be less prevalent in the grey matter of CCI rat spinal cords at earlier time points (4, 21 and 30 days; Figure 10B). The macrophage/activated microglia marker (CD11b-OX42) showed small, scattered cells of similar morphology to Cox-2 immunoreactive cells, usually with several processes (Figure 10C). These cells appeared to increase in number and intensity in the spinal cord on the side of the nerve lesion, mainly in the superficial dorsal horn (Laminae I-II) and the ventral horn (Laminae IX, Figure 10D).

**Figure 11:**
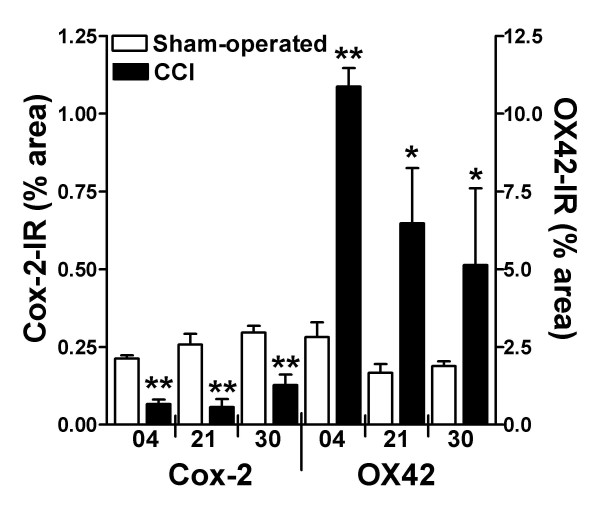
**Cox -2-IR and OX42-IR in dorsal horn of rat spinal cord (Laminae I–II). **Cox-2-IR levels were significantly lower in the lesioned superficial dorsal horn of the rat spinal cord compared to the sham-operated spinal cord at 4, 21 and 30 days post operation. OX42-IR was significantly increased in the lesioned superficial dorsal horn of the rat spinal cord across the time course. * p < 0.03; ** p < 0.02.

Quantification of Cox-2-IR in the time course showed significantly (p < 0.02) lower levels in the lesioned superficial dorsal horn of the spinal cord compared to sham-operated at all three time points (Figure 11). Quantification of OX42-IR showed increased significant levels of OX42-IR across the time course (p < 0.03) and across the main three areas in the lesioned side of the spinal cord – superficial dorsal horn (Figure 11), deep dorsal horn (data not shown) and ventral horn (data not shown).

## Discussion

Tissue damage generates an inflammatory response resulting in release of inflammatory mediators that in turn causes pain and hyperalgesia. Macrophages, other immunocompetent cells, as well as increased levels of cytokines have been found in injured nerves and DRG [35,38,39]. Macrophages have been found to be the predominant source of prostanoid release [40,41]. Prostanoids sensitise peripheral nerve terminals, and are also produced and released in the spinal cord following peripheral nerve injury, establishing both peripheral and CNS links between prostaglandin production and hypersensitivity [10,42].

In the present study, the time course of macrophage/microglia-like cell activation was studied in comparison with Cox-2 levels and EP1 receptor levels in human injured nerves and a rodent model of nerve injury. EP1 receptor levels were reported for the first time in human nerve fibres and DRG, and appeared increased in acutely injured tissues. The greater increase distally of EP1 was a trend, and not statistically significant, and could be the result of increased EP1 in regenerating or spared fibres. The EP1 time course when compared to the previously published time course of Cox-2 [35] showed rapid elevated levels of EP1 receptor in proximal nerves. The previous paper reported both proximal and distal nerve staining, but only proximal nerve stumps were used to compare directly with EP1 immunostaining for the same specimens in this study. In accord with our previous report [35], Cox-2-IR was increased from some weeks after injury, whereas CD68-positive macrophages were increased more acutely – in this study, we have demonstrated, in addition, that the Cox-2-IR increase in macrophages persists over many years in injured human neuromas. After injury nerves show rapid swelling and oedema formation. Due to this swelling, the % positive areas measured might account for the lower levels of Cox-2-IR with image analysis compared to controls – when we analysed our nerves by counting positive cell numbers per area, no significant change (i.e. no decrease) was observed. A 70 kDa Cox-2 band was significantly increased in acute injured human nerve extracts, but not chronic, and are apparently discrepant with immunohistochemical findings – this may reflect the time-points at which limited numbers of nerves were available in sufficient quantities to enable western blotting studies, as only those clustering around the broad peak of Cox-2 increase (2 – 8 weeks) would be expected, and showed, significant increase. The nerve samples extracts that constitute the Western blotting "acute" group represented the later stage of the acute phase i.e., 10, 15 and 17 days, and the "chronic" group included 3 out of 5 samples beyond 10 weeks after injury.

In the CCI rat model, numerous OX42-IR macrophage/microglial-like cells appeared in the injured sciatic nerves, as well as in the superficial dorsal horn of the spinal cord in CCI rats, at 4 days post operation. However, while there was a trend, Cox-2-IR was not significantly increased in nerves. A similar change was also found at 21 days post-operation in CCI nerve tissue in recent different set of animals (data not included); a significant increase of Cox-2-IR at 40 days post-injury in CCI nerve was reported by us previously [35]. In this study Cox-2 immunoreactivity just failed to reach significance at days 4 and 21 in the CCI rats, possibly because the increase was not as robust as at day 40 after injury, and/or due to a smaller number of animals in the present study. Two cited papers [43,44] showed an upregulation of Cox-2 in CCI and partial nerve ligation nerves at 2 and 4 weeks after injury, also in macrophages, but they counted Cox-2 positive cells per area in one study and a comparison between sham and CCI contralateral sciatic nerve was not assessed [43], whereas we image-analysed % area of the sections and conducted statistical comparison, which may account for the differences in our studies. When we re-analysed our CCI nerves by counting positive cell numbers per area, a trend for an increase was observed but this still did not achieve statistical significance (data not shown). In another study, when Cox-2 positive cells were compared between ipsi- and contralateral sciatic nerve (modified Chung model), not all time points (i.e. 3 days) reached levels of significance [45].

In the CCI rat spinal cord, a significant increase of OX42 immunoreactive macrophage/microglial-like cells was observed over the entire time course (4–30 days) in the lesioned side superficial dorsal horn; however, Cox-2-IR was, surprisingly, found to be decreased at all these time points. Sufficient tissues were not available for Western blotting of Cox-2 in rat spinal cord, but are necessary to substantiate present findings. The underlying mechanisms and significance of this decrease remain hence uncertain; it should be noted that we have previously reported using the same methods that Cox-2 immunoreactive macrophage-like cells in the nerves and in the lesioned superficial dorsal horn of the CCI rats were increased above normal levels at 40 days post operation, suggesting a delay in the expression of Cox-2 [35]. Structural reorganisation in the spinal cord after peripheral nerve injury [46] could account for the delayed expression of Cox-2 in macrophage/microglial-like cells.

A number of other studies have described Cox-2 changes in different cell types in animal models of nerve injury. Using a modified Chung model, where only L5 was severed [47,48], different stages of Cox-2 expression were observed in the sciatic nerve, with an early or first phase (after 1 day), where Cox-2-IR was co-localised with a Schwann cell marker, followed by a second phase, involving macrophages [45]. In the spared nerve injury (SNI) model [49], a small increase in Cox-2 mRNA protein was demonstrated in the dorsal horn at 24 hours post surgery, returned to sham levels at 72 hours, and was decreased at 7 days [50]. Furthermore, Cox-2-IR was shown by immunohistochemical methods to be only slightly increased in the deeper layers of the L4–L5 dorsal horn of the spinal cord at 10 hours post surgery. In this study pain behaviour in the rats was apparent from 9 days and maintained until 30 days post operation, which suggests that the neuropathic pain behaviour tested was not correlated, at the time-points studied, with significantly increased Cox-2 expression, in accord with the findings of some other investigators [32].

In a rodent partial nerve ligation study, EP1-IR was found near the sciatic nerve ligation site, in nuclei of cells co-expressing the macrophage marker ED1 [51]. However, in the present study, we found EP1 receptors to be mainly expressed in axons and cell bodies of human sensory neurones. The regulation of EP1 receptors in injured sensory neurones and inflammatory cells deserves further investigation. The molecular regulators of EP1 expression in DRG neurons are unknown – trauma and initial inflammatory response (shown by rapid increases of CD68-IR) may lead to increased EP1 levels, to which PGs may contribute. Later, Cox-2 expression in macrophages may be involved in the persistence of pain. The time-course of Cox-2 expression also suggests a role in the processes of Wallerian degeneration and regeneration. Further investigations are required, including studies of chronic non-painful human neuromas, to establish a link between EP1 and Cox-2 levels with pain.

A number of clinical and animal model pharmacological studies suggest that Cox-2 and EP1 are key therapeutic targets in inflammatory and neuropathic pain. The selective Cox-2 inhibitor, GW406381X, was shown to be effective in reducing mechanical allodynia in the CCI rat model, and thermal hyperalgesia in the mouse partial ligation model, both animal models of neuropathic pain [47]. GW406381X was also effective in reducing pain behaviour when given intrathecally and orally to rats with capsaicin-induced inflammatory pain [52]. The selective Cox-2 inhibitor, etodolac, administered orally, reduced heat-evoked hyperalgesia in rats with chronic constrictive sciatic nerve injury [53]. Other Cox-2 inhibitors such as celecoxib and rofecoxib, however, are effective in inflammatory pain but do not alter neuropathic pain behaviour. Intraperitoneal injection of Rofecoxib, a selective cox-2 inhibitor, did not prevent the development of allodynia and hyperalgesia in the spared nerve injury model [50].

EP1 antagonists may be effective in neuropathic, inflammatory and incisional pain models. In the CCI model, oral administration of an EP1 antagonist from 8 to 14 days post-operation effectively reduced CCI-induced mechanical hyperalgesia and allodynia [20]. In another animal model of neuropathic pain (partial ligation of the sciatic nerve), mechanical and thermal hyperalgesia was reversed with a EP1 receptor antagonist (SC-19220) [54]. EP1 receptor antagonists reduced the response to formalin-induced inflammation [55,56]. Spinal application of EP agonists in an inflammatory rat knee joint model demonstrated that EP1, EP2 and EP4 agonists all generated spinal hyperexcitability similar to PGE_2 _[57]. In a rat model of postoperative pain administration of an EP1 antagonist (ONO-8711) generated analgesic effects on mechanical evoked pain [58,59]. Similar inhibitory effects on mechanical hyperalgesia with the same EP1 antagonist were shown in a carrageenan-induced inflammatory model [60]. The second phase, but not the first phase, of formalin-induced flinching behaviour in the paw was effectively inhibited by spinally administered EP1 receptor antagonists (SC-51089 and SC-51234A) [56]. Importantly, the involvement of PGE2 in sensitisation via the EP1 receptor has also been shown in human oesophageal pain hypersensitivity, with the successful use of an EP1 antagonist [24].

## Conclusion

In this study, we report that a rapid increase of EP1 receptor levels in injured human sensory neurones preceded Cox-2 expression in infiltrating macrophages. Chronic painful human neuromas showed persistence of EP1-IR in nerve fibres and increased Cox-2-IR macrophages. EP1 antagonists may show therapeutic effects in acute and chronic neuropathic pain, in addition to inflammatory pain.

## Competing interests

The author(s) declare that they have no competing interests.

## Authors' contributions

Pascal F Durrenberger, Paul Facer and Maria Casula performed the immunostaining studies and helped write the manuscript. Roy A Gray, Sue D Collins, Sharon Bingham and Alex W Wilson prepared the CCI model and collected tissues. Iain P Chessell and Nicola C Day helped with the design of the studies, and the screening and selection of the antibodies. David Elliot and Rolfe Birch provided the human nerve specimens. Praveen Anand designed the study, helped collect the control nerve tissues, and helped write the manuscript.

## Pre-publication history

The pre-publication history for this paper can be accessed here:
